# A Rare Case of Solitary Brain Metastasis From Papillary Thyroid Carcinoma: The Role of Empiric I-131 Therapy and SPECT/CT (Single Photon Emission Computed Tomography/Computed Tomography) in Achieving an 11-Year Survival

**DOI:** 10.7759/cureus.105291

**Published:** 2026-03-16

**Authors:** Betul Vatankulu, Sabire Yilmaz

**Affiliations:** 1 Nuclear Medicine, Istanbul Aydin University VM Florya Medical Park, Istanbul, TUR; 2 Nuclear Medicine, Positron Imaging Center, Ankara, TUR

**Keywords:** brain metastasis, empiric therapy, i-131, papillary thyroid carcinoma, spect/ct, thyroglobulin

## Abstract

Brain metastases from papillary thyroid carcinoma (PTC) are rare and typically associated with a poor prognosis; however, early detection of solitary lesions can lead to exceptional long-term outcomes. We present the case of a 65-year-old man with a history of multicentric PTC (T1bN0M0, Stage I) treated with total thyroidectomy and postoperative radioiodine ablation 12 years earlier, who presented with rising thyroglobulin (Tg) levels (12 ng/mL) under TSH (thyroid-stimulating hormone) stimulation. Neck ultrasonography and 18F-FDG PET/CT (18-fluorodeoxyglucose positron emission tomography/computed tomography) failed to identify recurrent disease. Empiric high-dose (150 mCi; 5.55 GBq) I-131 therapy was administered. Post-therapy, whole-body imaging demonstrated focal cranial uptake, initially suspected as contamination. I-131 SPECT/CT (single photon emission computed tomography/computed tomography) fusion imaging localized intense iodine uptake to the right occipital lobe. A cranial MRI confirmed a 15 × 15 mm enhancing lesion. The patient underwent craniotomy, and histopathological evaluation confirmed metastatic PTC with follicular features. Immunohistochemistry was positive for TTF-1 and thyroglobulin, with a Ki-67 proliferation index of 20%. The patient remains clinically and biochemically stable and disease-free 11 years after the metastasectomy, demonstrating an exceptionally long survival period for brain metastasis from PTC. This case highlights the diagnostic value of empiric high-dose radioiodine therapy combined with SPECT/CT in detecting occult metastases. Furthermore, the 11-year disease-free survival in our patient underscores that early identification and surgical resection of solitary brain metastases from PTC can lead to an exceptionally favorable long-term prognosis.

## Introduction

In terms of global cancer incidence, thyroid cancer ranks seventh overall, while it represents the fifth most prevalent malignancy among women [1]. Papillary thyroid carcinoma (PTC) represents the most frequent thyroid cancer, exhibiting a favorable prognosis with a 10-year survival probability exceeding 90% [1,2]. While regional lymph nodes are the primary site of spread, the incidence of distant metastasis ranges between 3% and 10%, with the lungs and skeletal system being the most common sites. Conversely, involvement of the brain and other soft tissues is documented as an extremely infrequent clinical occurrence [3]. Brain metastases are rare, reported in 0.1-5% of patients, and are typically associated with disseminated disease and unfavorable outcomes [4].To date, there is no adequate knowledge regarding the survival benefit of radioiodine in patients with cerebral metastases, and evidence on such a rare metastatic presentation remains limited to case reports, series, and small-sample studies [5]. The detection of metastatic disease in patients with elevated thyroglobulin (Tg) levels but negative conventional imaging remains a clinical challenge. Although 18F-FDG PET/CT (18-fluorodeoxyglucose positron emission tomography/computed tomography) is frequently used in such scenarios, radioiodine imaging, particularly when combined with SPECT/CT (single photon emission computed tomography/computed tomography), may provide additional anatomical and functional information. We report a rare case of an asymptomatic solitary brain metastasis identified using empiric high-dose I-131 therapy followed by SPECT/CT imaging after negative ultrasonography and PET/CT findings.

## Case presentation

**Figure 1:**
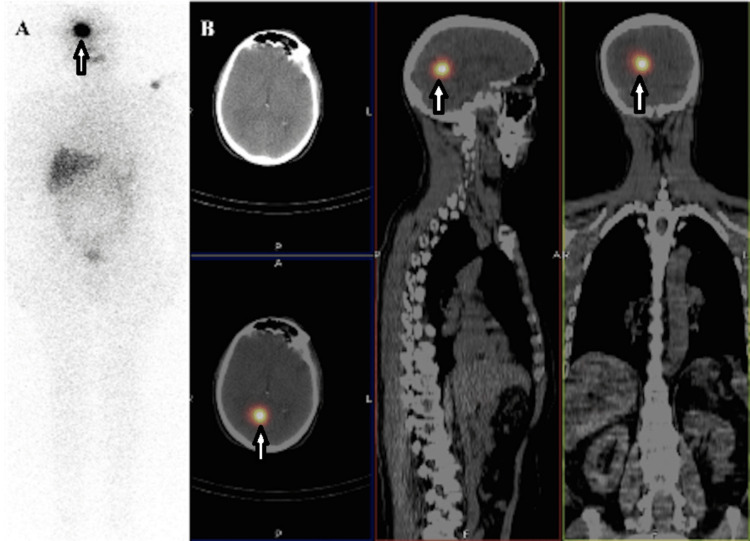
(A) Whole-body post-therapeutic I-131 scan demonstrating focal intense iodine uptake in the right frontal region of the cranium and the left shoulder (white arrows). (B) I-131 SPECT/CT fusion images confirming focal intense iodine uptake in the right occipital lobe (white arrows). The I-131 uptake on the left shoulder was evaluated as external contamination.

A 65-year-old male with a history of multicentric invasive PTC (T1bN0M0, Stage I) underwent total thyroidectomy followed by radioiodine ablation (100 mCi; 3.7 GBq) 12 years prior. Initial post-ablation whole-body scintigraphy showed no evidence of distant metastasis. The patient was classified as low risk according to the American Thyroid Association risk stratification system. During routine follow-up under TSH (thyroid-stimulating hormone) suppression therapy, serum Tg levels remained below 0.1 ng/mL for 12 years, with stable anti-Tg antibody levels. At the most recent evaluation, Tg levels increased under TSH stimulation (TSH 30 μIU/mL, Tg: 12 ng/mL), prompting further investigation. Neck ultrasonography revealed no pathological findings. 18F-FDG PET/CT also failed to demonstrate abnormal uptake. Due to persistent biochemical evidence of disease, L-thyroxine was discontinued to achieve TSH stimulation (TSH 100 mIU/L). Serum Tg increased to 48 ng/mL. Empiric radioiodine therapy (150 mCi; 5.55 GBq) was administered. Post-therapy, whole-body imaging demonstrated focal iodine uptake in the right cranial region and left shoulder. The shoulder uptake was considered contamination. SPECT/CT fusion imaging localized the cranial uptake to the right occipital lobe, confirming a true pathological focus (Figure 1).

**Figure 2:**
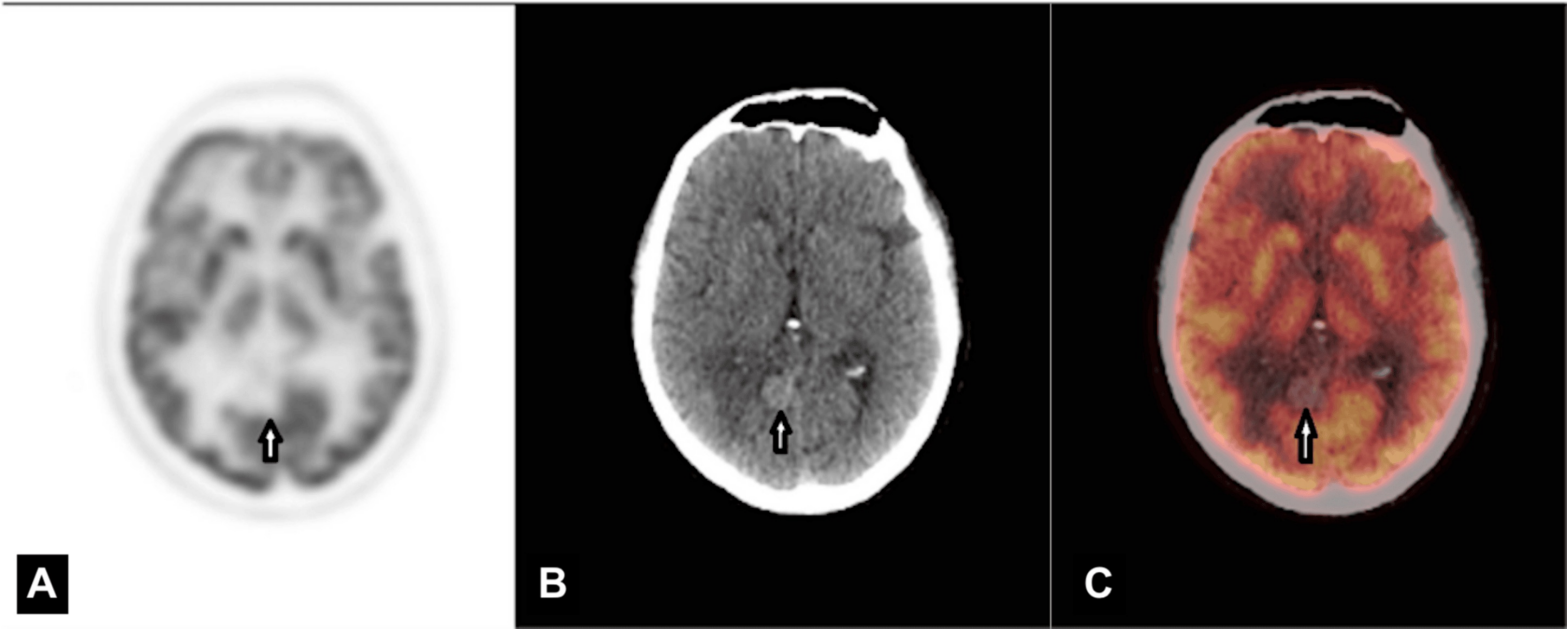
Axial FDG PET (A), CT (B), and fusion PET/CT (C) sections of the right occipital lesion demonstrating no significant FDG metabolic activity (white arrows), consistent with an iodine-FDG mismatch. FDG PET: fluorodeoxyglucose positron emission tomography; CT: computed tomography

A retrospective review of prior FDG PET/CT images revealed a subtle right occipital lesion without increased FDG uptake (Figure 2).

**Figure 3:**
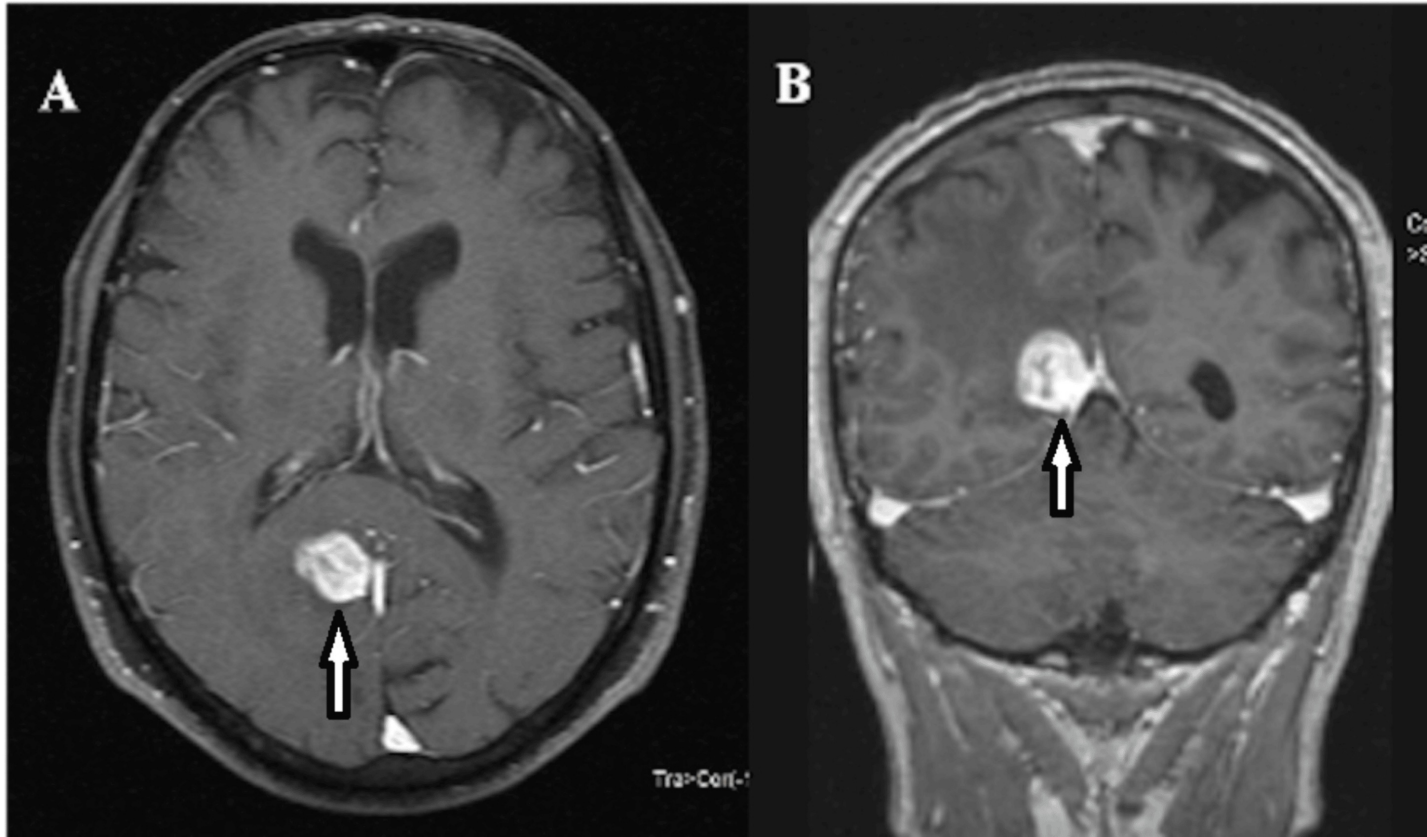
Axial (A) and coronal (B) post-contrast T1-weighted cranial MRI sections, revealing a 15x15 mm enhancing lesion in the right occipital region (white arrows), which corresponds to the previously identified iodine-positive focus.

A cranial MRI confirmed a 15 × 15 mm contrast-enhancing lesion in the right occipital region (Figure 3).

**Figure 4:**
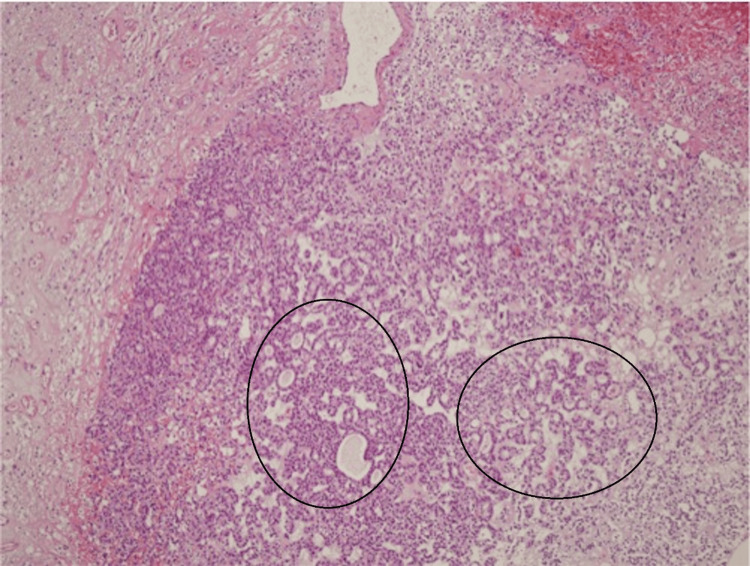
Microscopic appearance of the metastatic tumor demonstrating a predominantly follicular growth pattern (indicated by circles). Immunohistochemical analysis reveals strong HBME-1 and thyroglobulin positivity, confirming the thyroid origin of the metastasis (H&E, ×200). H&E: hematoxylin and eosin

The patient underwent surgical resection via craniotomy. Histopathological examination demonstrated metastatic thyroid carcinoma with predominantly follicular architecture. Tumor cells were strongly positive for TTF-1, thyroglobulin, and CK-56. The Ki-67 index was 20% (Figure 4).

The patient has remained under regular clinical and biochemical surveillance for 11 years following the metastasectomy. As of 2026, he remains asymptomatic and clinically stable under TSH-suppressive therapy. His serum Tg levels have remained undetectable, and periodic imaging has shown no evidence of local recurrence or new distant metastases. This remarkably long survival period confirms the success of the combined diagnostic and therapeutic approach.

## Discussion

Extensive observational evidence indicates a significant survival benefit from the use of adjuvant radioiodine in PTC management; notably, a recent 20-year study documented that the overall survival probability for patients undergoing RAI (radioactive iodine) treatment exceeds 95% [6]. However, brain metastases from differentiated thyroid carcinoma are uncommon and are typically associated with poor prognosis [2-4]. Most reported cases involve multiple distant metastatic sites. Solitary brain metastasis occurring more than a decade after initial diagnosis is exceedingly rare [7].

This case underscores several important points: First, biochemical recurrence may precede radiological detection. Rising Tg levels were the only indicator of metastatic disease. Second, FDG PET/CT may be negative in well-differentiated, iodine-avid cerebral metastases. Third, empiric high-dose radioiodine therapy (“shot-in-the-dark” approach) combined with SPECT/CT can improve lesion localization. Hybrid SPECT/CT imaging provides both anatomical and functional information, increasing diagnostic accuracy compared with planar scintigraphy alone [8]. In this patient, SPECT/CT precisely localized the lesion, guiding surgical management. The Ki-67 index of 20% suggests increased proliferative activity and may correlate with a more aggressive biological behavior, despite the well-differentiated histology. The prognosis for patients presenting with cerebral metastases from thyroid carcinoma is generally poor, with recent literature indicating a median survival of only 15 months following the diagnosis [6]. Factors such as younger age, solitary lesions, and surgical resectability have been shown to improve outcomes [3]. Our case is particularly noteworthy for the 11-year recurrence-free survival following the detection of an asymptomatic lesion. This highlights that a high index of clinical suspicion, prompted by rising Tg levels despite negative conventional imaging, can lead to life-saving interventions.

## Conclusions

This case demonstrates that even in the absence of findings on conventional imaging, rising thyroglobulin levels should be investigated with empiric high-dose I-131 therapy and SPECT/CT. The identification and subsequent surgical resection of a solitary, asymptomatic brain metastasis in our patient resulted in an 11-year disease-free survival. This underscores the importance of a high clinical suspicion and a multimodality imaging approach in achieving long-term survival in patients with differentiated thyroid carcinoma.
